# Editorial: Bildung für eine digitale Zukunft

**DOI:** 10.1007/s11618-021-01049-w

**Published:** 2021-09-29

**Authors:** Katharina Scheiter, Ingrid Gogolin

**Affiliations:** 1 Arbeitsgruppe Multiple Repräsentationen, Leibniz-Institut für Wissensmedien, Universität Tübingen, Schleichstraße 6, 72076 Tübingen, Deutschland; 2 Fakultät für Erziehungswissenschaft, Allgemeine, Interkulturelle und International Vergleichende Erziehungswissenschaft, Universität Hamburg, Von-Melle-Park 8, 20146 Hamburg, Deutschland

Das Thema Digitalisierung hat – nach einem langen Dornröschenschlaf – das deutsche Bildungswesen erreicht. Meilensteine dieser Entwicklung umfassen unter anderem die Veröffentlichung eines Strategiepapiers der Kultusministerkonferenz (2016) zur ‚Bildung in einer digitalen Welt‘, in der Ziele und Handlungsbedarfe für unterschiedliche Bildungsakteure beschrieben werden; die 2017 begonnene Strategie des Bundesministeriums für Bildung und Forschung (BMBF) zur Förderung von Untersuchungen zur Digitalisierung im Bildungsbereich; die Erweiterung der Qualitätsoffensive Lehrerbildung des BMBF um einen Förderschwerpunkt Digitalisierung (2018), in dessen Rahmen digitalisierungsbezogene Konzepte und Angebote für die universitäre Ausbildung angehender Lehrkräfte entwickelt werden sollen sowie die Verabschiedung des DigitalPakts des Bundes und der Länder (2019), mit dem an Schulen die technischen Voraussetzungen für einen mediengestützten Unterricht geschaffen werden sollen.

Diese Maßnahmen trafen in Deutschland auf eine schwierige Ausgangslage: Digitalisierungsbezogene Kompetenzen von Schülerinnen und Schülern sowie Lehrkräften ebenso wie die Mediennutzung im Unterricht liegen laut Ergebnissen der International Computer and Information Literacy Studies (ICILS) von 2013 und 2018 im internationalen Vergleich teilweise deutlich unter dem OECD-Durchschnitt (Bos et al. 2014; Eickelmann et al. 2019). In der ersten Phase der Ausbildung von Lehrkräften ist das Thema Digitalisierung an den wenigsten lehrerbildenden Standorten sichtbar und nachhaltig verankert. In den wenigen Hochschulen, in denen es überhaupt vorkommt, beschränkt es sich auf nicht-verpflichtende Lehrangebote (Monitor Lehrerbildung 2020).

In den meisten Schulen erlaubte zudem die technische Ausstattung keinen didaktisch sinnvollen Einsatz digitaler Medien im Unterricht. Das Angebot an digitalen Lehr-Lernmaterialen ist überschaubar und vielfach ohne Berücksichtigung der Potenziale digitaler Medien entwickelt.

Auf diese Situation trafen im März 2019 die ersten pandemiebedingten Schulschließungen. Über Nacht wurden Alternativen für den Präsenzunterricht erforderlich. Die bis dahin nur sporadisch erfolgte Nutzung digitaler Medien wurde jetzt notwendig für ein Lehren und Lernen auf Distanz – Unterrichten per Videokonferenz, Austausch von Materialien und Aufgaben per Email und Cloud, individuelle Sprechstunden per Chat, etc. Wie inzwischen aus diversen Umfragen bekannt, erfolgte die Umsetzung an Schulen in Deutschland sehr unterschiedlich – sowohl hinsichtlich des Umfangs als auch der Vielfalt und Innovation der eingesetzten didaktischen Möglichkeiten (Helm et al. 2021; Vodafone Stiftung Deutschland 2020). Insgesamt zeigte sich, dass in Deutschland großer Aufholbedarf bei der Vorbereitung und Umgestaltung des Bildungssystems für eine digitale Zukunft besteht (vgl. auch weitere Beiträge im Themenheft 2‑2021 der ZfE).

Eine wesentliche Ressource – neben der Bereitstellung der notwendigen technischen Infrastruktur – besteht in wissenschaftlichen Erkenntnissen, die es erlauben, die Vielfältigkeit der Herausforderungen aus einer theoretischen Perspektive zu beschreiben und Maßnahmen zur Unterstützung der digitalen Transformation auf unterschiedlichen Ebenen forschungsbasiert zu entwickeln.

Dies betrifft zum einen die didaktische Gestaltung und Bereitstellung digitaler Bildungsressourcen. Wissenschaftliche Erkenntnisse zur Lernwirksamkeit digitaler Lernwerkzeuge zeigen inzwischen übereinstimmend, dass digitale Medien bzw. ihre medienspezifischen Merkmale (z. B. Multimedialität, Adaptivität) Lernergebnisse substanziell verbessern können (z. B. Hillmayr et al. 2020). In den entsprechenden Studien umgesetzte, lernwirksame Designmerkmale digitaler Medien sind in der empirischen Lehr-Lernforschung seit den 1970er-Jahren bekannt, finden aber nur sporadisch Einzug in die Praxis (Scheiter und Lachner 2019). Scheiter diskutiert darüber hinaus in ihrem Stichwortbeitrag, dass die entsprechende Forschung sich vielfach auf die Gestaltung einzelner Lernmedien konzentriert hat, die von Lernenden eigenständig und häufig als Ersatz für das Lehrangebot einer Lehrperson genutzt werden sollen. Dabei bleiben die Fragen nach einer sinnvollen Integration digitaler Lehr-Lernangebote in den Unterricht und der Verknüpfung dieser Angebote mit anderen – analogen – Unterrichtspraktiken außen vor. Entsprechend werden in dem Beitrag von Scheiter zwei Forschungsperspektiven – *technology-enhanced learning* und *technology-enhanced teaching* – beschrieben und zur Herleitung von Forschungsdesiderata herangezogen. Wesentlich ist die Forderung nach einer stärkeren Verbindung der beiden Perspektiven. Die Autorin diskutiert spezifische Medienmerkmale im Hinblick auf ihr Potenzial, lernwirksamen Unterricht zu realisieren, dessen prinzipielle Eigenschaften in der Unterrichtsforschung gut beforscht sind (Kunter et al. 2013). Daran schließt sich beispielsweise die Forschungsfrage an, wie digitale Medien gestaltet und eingesetzt werden können, damit sie zur kognitiven Aktivierung oder zur konstruktiven Unterstützung von Schülerinnen und Schülern beitragen können.

Ein wesentlicher Vorteil digitaler Bildungsressourcen liegt darin, dass diese beliebig für unterschiedliche Zielgruppen bereitgestellt und uneingeschränkt genutzt werden können. Sogenannte *Open Educational Resources* (OER) bieten die Möglichkeit, den Arbeitsaufwand und die Kosten bei der Erstellung von Bildungsangeboten für die einzelne Nutzung zu reduzieren, so dass auf eine – zu kommerziellen Angeboten alternative – Auswahl von Lehr-Lernmaterialien zurückgegriffen werden kann. Otto et al. beleuchten im Rahmen einer systematischen Mapping-Studie den internationalen Forschungsstand zu OER, der sich bislang auf den Hochschulsektor konzentriert. Adressiert wird vor allem die Wahrnehmung von OER durch potenzielle Nutzer und Nutzerinnen. Es überwiegen Studien zur Begutachtung existierender OER-Angebote. Ableitungen von Handlungsempfehlungen, Theoriebildung und Arbeiten zu Implementierungsstrategien sind eher selten. Otto et al. bewerten die empirische Forschung zu OER als in den Anfängen begriffen.

Wesentlich für ein Gelingen des digitalen Transformationsprozesses sind – neben der Verfügbarkeit digitaler Bildungsressourcen – entsprechende Kompetenzen des pädagogischen Personals, und hier insbesondere der Lehrpersonen. Dabei verweist die Forschung, wie von Scheiter diskutiert, auf die Notwendigkeit eines breit angelegten Kompetenzbegriffs, der *sensu* Weinert (2001) sowie Baumert und Kunter (2006) nicht nur Wissen und Fertigkeiten, sondern auch Motivation, Einstellungen und selbstregulative Aspekte umfasst. Vor allem im angloamerikanischen und asiatischen Raum wird relevantes Wissen für das Unterrichten mit digitalen Medien vor dem Hintergrund des so genannten TPACK-Modells von Mishra und Koehler (2006) konzipiert. Danach müssen klassische Wissenskomponenten, wie sie bereits von Shulman (1987) beschrieben wurden – pädagogisches Wissen, fachliches Wissen und fachdidaktisches Wissen – für das Unterrichten mit digitalen Medien um technologisches Wissen ergänzt werden. An der Schnittstelle dieser Wissensbestände ist technologisches-pädagogisches Inhaltswissen (*technological pedagogical content knowledge*) angesiedelt, welches Lehrende befähigt, digitale Medien in didaktisch sinnvoller Weise für das Erreichen fachlicher Bildungsziele einzusetzen. Bürger et al. arbeiten in ihrem Beitrag hinaus, dass neben dieser wissensbezogenen Komponente Einstellungen und Motivation wesentlich für die Nutzung digitaler Technologien im Unterricht sind (siehe auch Scherer und Teo 2019). Bürger et al. liefern einen Überblick über den – hinsichtlich der verwendeten Forschungsmethoden und -instrumente heterogenen – Stand der Forschung und systematisieren die Befunde aus 74 Studien vor dem Hintergrund der *Theory of Planned Behavior*. Positive Einstellungen gegenüber digitalen Medien sowie eine hohe Motivation erweisen sich in der überwiegenden Zahl der Studien als Prädiktoren für die Intention, digitale Medien im Unterricht einzusetzen, und für deren faktische Nutzung. Erkenntnisse zum Zusammenhang von Einstellungen und Motivation und der Qualität des Unterrichts fehlen dagegen mit wenigen Ausnahmen (z. B. Backfisch et al. 2020, 2021).

Ein umfassendes Verständnis der notwendigen Kompetenzen für das Unterrichten mit digitalen Medien ist wesentlich für die Lehrerbildung (Caena und Redecker 2019). Auf der Mikroebene könnte eine umfassende Konzeption der digitalisierungsbezogenen Kompetenzen von Lehrkräften die unterrichtliche Praxis informieren und als Zielgröße für eine – an die Bedürfnisse angepasste – berufliche Aus- und Weiterbildung der Lehrkräfte fungieren. Auf der Mesoebene könnte sie die Bildungspolitik unterstützen, indem sie z. B. Informationen für die Entwicklung von Schulen und Lehrerbildungsprogrammen liefert. Auf der Makroebene könnten sie durch die Bereitstellung evidenzbasierter Referenzstandards für die Lehrerausbildung eine Qualitätssicherung ermöglichen. Schulze-Vorberg et al. Zeigen in einer Befragung von 238 Gymnasiallehrkräften, dass diese je nach Ausprägung ihrer digitalisierungsbezogenen Kompetenzen unterschiedliche Präferenzen für Fort- und Weiterbildungsangebote aufweisen, die auf die Notwendigkeit einer bedarfsgerechten Gestaltung derselben hindeuten. Diese Gestaltung adaptiver Angebote könnte eine Möglichkeit sein, der bislang beobachtbaren geringen Bereitschaft von Lehrkräften zur Teilnahme an digitalisierungsbezogenen Maßnahmen entgegenzuwirken (Gerick et al. 2019). Die Ergebnisse von Schulze-Vorberg et al. legen nahe, dass hier insbesondere die Gruppe der Abseitsstehenden, d. h. derjenigen Lehrkräfte, die eher geringe wissensbezogene und motivationale digitalisierungsbezogene Kompetenzen aufweisen, in den Blick genommen werden sollte, da diese bislang nur an selten an thematisch passenden Veranstaltungen teilnehmen. Schulze-Vorberg et al. diskutieren Implikationen ihrer Arbeit vor allem im Hinblick auf die Gestaltung entsprechender Fort- und Weiterbildungsangebote.

Mit den Beiträgen zum Themenheft wird der erreichte Stand von Forschung über Bildung für eine digitale Zukunft in einigen Facetten beleuchtet – zugleich aber auch verdeutlicht, dass es erheblichen weiteren Forschungsbedarf gibt. Dieser betrifft sowohl die Entwicklung und wissenschaftliche Prüfung adäquater Ansätze und Methoden für den Einsatz digitaler Medien in der Bildungspraxis selbst als auch das Wissen über angemessene Wege der Qualifikation des Personals, die eine wesentliche Voraussetzung für die Implementation entsprechender Ansätze wären. Die Beiträge zum Thema bieten eine Momentaufnahme. Es ist damit zu rechnen, dass – angestoßen durch die notwendigen Reaktionen auf die COVID-19-Pandemie – eine Beschleunigung der Forschung und Entwicklung stattfindet. Die ZfE wird also das Thema nicht zum letzten Mal ins Zentrum einer Ausgabe gestellt haben …
